# The Newer Remedies

**Published:** 1899-11

**Authors:** 


THE NEWER REMEDIES. A Reference Manual for Physicians, Pharmacists and Students. By VIRGIL COBLENTZ, A. M., Phar. M., Professor of Chemistry and Physics in the New York College of Pharmacy. Third edition, revised and enlarged; 150 pages. Philadelphia: P Blakiston's Son & Co., 1012 Walnut Street. 1899.

The physician who would keep up with modern materia medica in these days when chemistry is making almost daily additions to our armamentarium finds this manual indispensable. The subjects are arranged in alphabetical order, and a concise description of the remedy, its source, appearance, properties, and therapeutic use, is given.

